# “Cause they’re girls/boys”: preschool children’s play and toy choices

**DOI:** 10.3389/fpsyg.2025.1650155

**Published:** 2025-09-30

**Authors:** Hurşide Kübra Özkan-Kunduracı

**Affiliations:** Department of Child Development, Faculty of Health Sciences, Ankara Yıldırım Beyazıt University, Ankara, Türkiye

**Keywords:** gender, children’s play, toys, play motivation, gender identity

## Abstract

This case study aims to determine children’s play and toy preferences that they think are specific to girls and boys. The study group consisted of 50 children (21 girls, 29 boys) aged 48–72 months, attending a preschool in İstanbul, Türkiye. The data were obtained through pictures and semi-structured interviews and evaluated through descriptive analysis. As a result, the opinions on the plays by both girls and boys were mostly gathered under “pretend play.” Children included the physical movement type of play in a minority of the girls’ plays. In addition, children expressed their opinions mostly in the “representative toys” category. On the other hand, differences were observed in the types of representative toys in the girls’ and boys’ toys. While boys’ toys in this category were frequently cars, imaginary heroes, and army toys; girls’ toys were frequently dolls, character-figure toys, and doll houses. As another result, both girls and boys mostly reported gendered expressions and expressions of emotions regarding the motivations for the plays by children. In line with these results, both girls’ and boys’ views on plays and toys differ depending on gender but, both girls’ and boys’ views are parallel to each other.

## Introduction

Gender encompasses the characteristics associated with being male or female. During the preschool years, children increasingly exhibit behaviors that align with culturally prescribed gender roles. This period is marked by a strengthening of gender-specific behaviors and preferences among both girls and boys. Play, in particular, plays a crucial role in the socialization process related to sexual development (Frost et al., 2008). Boys and girls often display markedly different play behaviors (Trawick-Smith, 2014), and these differences are well-documented in the psychological literature. Such differences manifest in various aspects of play, including toy preferences, play styles, and peer interactions. These gendered play behaviors can have significant implications for the physical, social, and cognitive development of children, potentially leading to broader gender differences in developmental outcomes (Weisgram, 2022).

Research consistently indicates that gender differences in play are evident across all socioeconomic groups (Trawick-Smith, 2014) and cultures (Frost et al., 2008). The initial differences manifest in the visual preferences observed in infants. Alexander et al. (2009) conducted a study utilizing eye-tracking technology to investigate the visual preferences of 30 infants aged three to eight months regarding a doll and a truck. The findings revealed significant sex differences in visual interest in gender-associated toys. Specifically, female infants demonstrated a greater visual preference for the doll than the toy truck. In contrast, male infants exhibited a higher frequency of visual fixations on the truck compared to their female counterparts (Alexander et al., 2009). The differences in visual fixation duration persist from infancy into toddlerhood (Jadva et al., 2010). Gender differences become evident from the first year of life, so much so that children show distinct preferences for toys, such as toy cars versus dolls (Boe and Woods, 2018; Jadva et al., 2010; Lauer et al., 2018). Between the ages of two and three years, children increasingly engage in imaginative play, and gender-specific patterns become more pronounced. Boys tend to gravitate toward more active, rough-and-tumble play, while girls often prefer quieter, more passive activities. For instance, girls may engage in nurturing behaviors like singing lullabies to dolls, whereas boys are more inclined toward physically active play such as running with stick horses (Frost et al., 2008; Maccoby, 1998). Meta-analytic and large-scale studies have demonstrated that these gender differences in play behavior are substantial and tend to increase with age (Golombok and Rust, 1993; Golombok et al., 2008; Davis and Hines, 2020). Because gender stereotypes have better understood and stabilized over time. By the age of 3, children exhibit a significant understanding of stereotypes, leading to a noticeable increase in gender rigidity, especially by age 4 (Halim et al., 2013, 2014). At the same time, their flexibility in stereotypes declines until around age 7 (Banse et al., 2010).

Preschool children associate various items—such as clothing, tools, household objects, occupations, colors, and behaviors—with gender. These associations influence not only their play preferences but also their actions, reflecting their beliefs about gender roles. Boys are often observed to be more active, impulsive, assertive, and physically aggressive, whereas girls tend to exhibit traits such as timidity, dependence, emotional sensitivity, passivity, and a greater tendency toward indirect relational aggression (Berk, 2013). During early childhood, these gender-based beliefs become more entrenched, with many children perceiving them as rigid rules rather than flexible guidelines (Berk, 2013). Additionally, children at this stage predominantly choose to play with peers of the same sex, which reinforces gender-specific behaviors. Girls who prefer same-sex playmates are generally less active and more likely to remain close to adults. In contrast, boys are often more aggressive in their play and exhibit a preference for activities that are less supervised by adults (Blatchford, 1998; Boyatzis et al., 1999; Maccoby, 1998; Martin and Fabes, 2001).

Children’s play has significant socialization effects on sexual development, demonstrating considerable variation across different cultures. Key factors such as family dynamics, peer interactions, and mass media play critical roles in shaping gender differences in children’s play behaviors (Frost et al., 2008; Garvey, 1990). In addition to the influence of family, peers, and media, children’s gendered play behaviors can also be understood through broader developmental and socialization processes. One of these processes is gender-typing, defined as the acquisition of thoughts and behaviors aligned with culturally defined gender roles (Leaper and Bigler, 2018; Turner and Gervai, 1995). Gender-typing is shaped by both individual factors, such as cognitive-developmental mechanisms, and environmental influences (Endendijk et al., 2018; Kislev and Saguy, 2025). Parental influence is particularly critical and is often described as gendered parenting. This concept refers to parents’ tendency to consciously or unconsciously transmit gendered expectations through both direct reinforcement and more subtle practices such as the use of gendered language (Aznar and Tenenbaum, 2015; Endendijk et al., 2018; Mesman and Groeneveld, 2017; Morawska, 2020). Through play, children internalize societal norms and dominant gender beliefs imparted by adults, which leads them to conform to culturally prescribed gender roles. As a result, their behaviors frequently align with cultural stereotypes and norms (Yuen and Shaw, 2003). Interestingly, research has shown that at 12 months of age, both girls and boys tend to look longer at dolls compared to their attention at 18 or 24 months (Jadva et al., 2010). This observation suggests that the aversion to dolls often seen in older boys may develop later in their lives. Consequently, the gender differences observed in preferences for toys, colors, and shapes during later developmental stages may be attributed more to socialization processes and cognitive gender development than to inherent or congenital factors (Jadva et al., 2010). Moreover, the developmental intergroup theory proposed by Bigler and Liben (2007) provides a robust framework for understanding the causal factors underlying stereotyping and prejudice. This theory posits that gender biases in children’s play and toy preferences are significantly influenced by educational, social, and legal policies, underscoring the complexities of gender socialization. This research was conducted in Turkey, which has distinct educational, social, and legal policies. The country also has diverse cultural backgrounds that influence stereotypes and prejudices. As a result, it is essential to understand the game and toy preferences of Turkish children.

## Current study

Research has highlighted significant differences between boys’ and girls’ play behaviors, including variations in toy preferences (Alexander et al., 2009; Boe and Woods, 2018; Jadva et al., 2010; Lauer et al., 2018). These gender differences in play preferences, such as the types of toys favored, are well-documented (Scapellato and De Pedis, 2021). Recent studies indicate that while boys and girls exhibit similar patterns of gender differences in play behaviors and toy choices, these differences become more pronounced with age. Additionally, environmental factors, particularly parental behavior, play a significant role in shaping these gender-specific preferences (Davis and Hines, 2020). To accurately assess gender differences in play, it is essential to consider observations from children’s natural environments rather than relying solely on structured settings (Göncü, 2001; Göncü et al., 2000). Moreover, the research literature on children’s toy preferences employs four primary methodological approaches: free play, visual preference, forced choice, and naturalistic observation. In free play studies, children are given a selection of toys and are allowed to interact with them in an unstructured manner. Visual preference paradigms involve presenting children with toys or images of toys, either sequentially or simultaneously, to assess their preferences. Forced-choice studies require children to select between two toy options presented by the experimenter, typically contrasting toys associated with male and female stereotypes. Naturalistic studies aim to minimize the experimenter’s influence on both the stimuli and the observed behaviors, thereby assessing toy preferences in a more organic context without predetermined toy selections (Davis and Hines, 2020). Beyond that, allowing children to express their views in unstructured environments, and through symbolic methods such as photography and drawing, provides valuable insights (Clark, 2005; Clark and Moss, 2011; Temel et al., 2018). Children’s drawings serve as visual data that can reveal how they perceive and interpret their experiences. These drawings reflect children’s feelings and thoughts symbolically, offering practical tools for evaluating their perceptions (Barraza, 1999; Einarsdóttir, 2007; Malchiodi, 2013). Furthermore, understanding how children perceive their drawings is crucial; engaging them in discussions about their drawings can enhance the clarity of insights into their experiences and thoughts (Malchiodi, 2013).

This naturalistic study utilizes both interviews and children’s drawings to gain a more nuanced understanding of play and toy preferences. Incorporating these methods aims to provide more comprehensive and accessible insights into children’s perspectives. It is essential to explore how children perceive and explain differences in play and toy choices, including their reasons for these preferences, to effectively monitor and evaluate these behaviors during early childhood. Identifying stereotypical gender labels associated with play and toy preferences for both same-gender and cross-gender activities will enhance our understanding of children’s play behaviors. Therefore, this study investigates children’s views on play and toy choices related to their gender and the opposite gender. The specific objectives of this study are to examine the play and toy choices of children aged 48–72 months, with a focus on gender differences. To achieve this, the study seeks to address the following research questions:

- How do girls and boys describe play activities?
- What toys do girls prefer, and what toys do boys prefer?
- What are children’s views on the motivations behind the play preferences of both girls and boys?

## Method

### Study design

In this study, a case study design, which is a qualitative research approach, was employed to examine the perspectives of preschool children regarding play activities specific to their own gender and the opposite gender. In case studies, the factors related to a given situation are investigated with a holistic approach, focusing on how these factors affect the situation or how they are influenced by it (Yıldırım and Şimşek, 2016). The most distinctive feature of case study research is the delimitation of the subject of the study. Such research represents not only a choice of what will be investigated, but also a methodological decision, since the focus of inquiry is a bounded system (Merriam, 2015).

According to Patton (2014), case studies may involve examining different groups or phenomena. In the present study, the “case” under investigation is the perceptions and viewpoints of preschool children regarding gender-specific play. Therefore, as emphasized by Patton (2014), these views were treated as the primary unit of analysis. Creswell (2014) also defines case study as a qualitative approach in which the researcher explores one or more bounded cases in depth over time through detailed data collection from multiple sources and reports a comprehensive description of the case.

In case study research, multiple data collection methods are generally used to obtain rich and reliable data. Accordingly, in this study, in line with the nature of the qualitative case study design, both semi-structured interviews and the drawing technique were employed. Along with the interviews conducted with the children, their play-themed drawings were also evaluated as data sources, thereby ensuring data triangulation. This approach enhanced the trustworthiness of the research (Merriam, 2015).

### Participants

The study group comprised 50 children (21 girls and 29 boys) aged between 48 and 72 months, attending a public preschool in Istanbul during the fall term of 2024. Of these, eight children were between 48 and 57 months old, while 42 were between 58 and 72 months old. The participants were selected using a convenience sampling method, which is appropriate when subjects are readily available, easily accessible, and willing to participate in the research. Since the study employed a qualitative methodology, no prior statistical power analysis was conducted to determine the number of participants. Instead, the sample size was determined by the principle of data saturation, a widely accepted approach in qualitative research. Data saturation is reached when similar responses and themes begin to recur across participants, indicating that further data collection is unlikely to yield new insights. In the present study, interviews continued until the researchers determined that participants’ perspectives had been sufficiently and comprehensively captured (Creswell, 2014; Merriam, 2015).

### Instruments

In qualitative research, data can be collected through various methods such as observations, interviews, documents, and audio-visual materials, each with its own strengths and limitations. The aim of this study was to explore the perspectives of children aged 48–72 months regarding their own and the opposite gender’s play and toy preferences. However, since children’s views on the opposite gender could not be directly observed and considering the possibility that they might perceive the researcher as an uninvited guest and thus refrain from displaying their natural behaviors, data were collected using developmentally appropriate techniques such as semi-structured interviews and drawing activities instead of direct observation. These methods enabled children to express their perspectives in ways suited to their developmental level and provided rich insights into their perceptions (Creswell, 2014; Einarsdóttir, 2007).

#### Instructions for drawing pictures

The drawing technique is extensively utilized in education and psychology to gain insights into children’s emotions and thoughts. Children often convey their feelings and perceptions symbolically through their drawings. The quality of the lines and the content of the drawings can reveal children’s attitudes and perceptions about themselves, their surroundings, and the individuals around them (Sayıl, 2004). Research suggests that children frequently express their feelings and thoughts more effectively through drawings than through verbal communication (Cherney et al., 2006). In this study, the drawing technique was employed to gather data. Children were asked to create drawings in response to two prompts: *“Draw the types of play that girls engage in the most”* and *“Draw the types of play that boys engage in the most.”* No time limit was imposed for completing their drawings. Once each child had finished, they were asked to describe their drawings and explain their thoughts. These verbal explanations were recorded on the back of each drawing to provide additional context for the visual data.

#### Interview

Interviews are a widely used data collection method in qualitative research, valued for their ability to elicit detailed information about aspects of the research subject that are not directly observable, and for their capacity to allow for alternative explanations (Glesne, 2015; Merriam, 2015). This study employed semi-structured interviews, which provided the researcher with the flexibility to explore additional relevant topics and obtain in-depth insights (Merriam, 2015). The objective of the semi-structured interviews was to investigate children’s perspectives on play activities typically associated with boys and girls. To ensure the effectiveness of the interviews as a data collection tool, a semi-structured interview form was developed by the researchers. This form was designed to facilitate the collection of comprehensive and relevant data. To ensure content validity, the draft interview form was reviewed by three experts: a faculty member with specialized knowledge in the field and two experts with advanced degrees in “play development.” The form was revised based on their feedback and finalized accordingly. The interview form comprised three core questions:

1. “Which types of play do boys/girls engage in?”
2. “What toys do boys/girls use?”
3. “Why do you think boys/girls engage in these types of play?”

Additional questions were included as needed to probe further into the subject matter.

### Procedures

This research was conducted in two distinct phases. In the initial phase, all necessary permissions were obtained following with ethical guidelines. In the subsequent phase, the implementation schedule was carefully planned to avoid disrupting the school’s regular activities. Before the main study, one of the researchers conducted introductory sessions in the classrooms. During these sessions, the researcher introduced herself, engaged in play with the children, and explained the purpose of the forthcoming study. Following these preparatory activities, the classroom was organized for the research implementation. A4-sized paper was provided for each child, along with an assortment of dry color pencils, felt-tip markers, and pastel paints in a variety of colors, to facilitate their drawing.

Drawing activities were conducted over two separate days. On the first day, all children were asked to “draw the types of play that girls engage in the most.” The children were given as much time as needed to complete their drawings. During this process, the researcher took notes and recorded observations of the figures drawn by the children. The researcher also engaged with the children by asking questions about their drawings and noting their responses on the back of each drawing. This approach enhances the clarity and effectiveness of the drawing technique (Einarsdóttir, 2007). Following the drawing session, the researcher initiated individual interviews with the children in a conversational and relaxed setting. The children were asked to elaborate on their drawings and provide insights into girls’ play activities. The researcher used probing questions to encourage more detailed responses. On the second day, the same process was repeated with the instruction to “draw the types of play that boys engage in the most,” again without imposing a time limit. After completing both sessions, the researchers provided small gifts to the children as a token of appreciation and thanked them for their participation in the study.

### Data analysis

Descriptive analysis was employed to evaluate the children’s drawings and interview responses. A data analysis framework was established based on relevant literature. Categories were developed to classify the types of play and toys preferred by the children (Yıldırım and Şimşek, 2016). As Merriam (2015) emphasizes, what defines a case study is not merely the research topic itself but the unit of analysis that guides the inquiry. In this study, the unit of analysis was the children’s perceptions and viewpoints regarding gender-specific play activities. Similarly, Stake (2006) highlights that cases must be understood as bounded systems, which require the researcher to focus within certain limits. Accordingly, this study focused on the bounded system of preschool children in a specific educational context and their play experiences.

The data analysis process was also carried out in line with the characteristics of case study research, which involves integrating and comparing multiple sources of evidence to capture the complexity of the phenomenon studied (Merriam, 2015). In this study, interview data and children’s drawings were evaluated comparatively and synthesized under common themes, ensuring a holistic perspective. The children’s preferred play activities were organized into four categories: “pretend play,” “physical-movement play,” “tabletop play,” and “games with rules” (Frost et al., 2008). However, the “games with rules” category was excluded from the analysis tables due to the developmental characteristics of the 5 to 6-year-old participants. At this developmental stage, children typically struggle with games that require adherence to rules without adult assistance, owing to factors such as egocentrism and difficulty in taking turns. The children’s toy preferences were categorized into four main types based on the frameworks established by (as cited in Van Hoorn et al., 2007). The categories used were: sensory-motor toys, representational toys, construction toys, and movable ride-on toys.

- *Sensory-motor toys* included items that stimulate the senses and motor skills, such as bouncing balls, shaking rattles, spinning balls, and rocking horses.
- *Representational toys* encompassed items that resemble real-life objects, such as miniature animals, toy vehicles, dollhouses, tools, furniture, and dolls.
- *Construction toys* are those that can be manipulated to create new structures or objects, including building blocks, wooden blocks, LEGO bricks, and tin toys.
- *Movable ride-on toys* consist of items that children can ride, such as bicycles, skateboards, and ride-on cars.

Additionally, since children’s responses regarding toys often included references to tabletop play games and physical-movement toys, these categories were also incorporated into the analysis tables to provide a comprehensive overview of the children’s toy preferences.

The categories related to the motivations behind girls’ and boys’ play were developed using the dimensions outlined in the processes of play as described by Russ (2004) and the Affect in Play Scale (Russ, 1987; Russ, 1993). These frameworks informed the categorization of play motivations into three categories: Expression of emotion, enjoyment of play, and gender discourses.

After establishing the categories, the children’s drawings (a total of 100) were initially pre-screened. Two researchers assessed whether the drawings adhered to the given instructions. Drawings from children coded F6 and M7 were excluded from the evaluation of girls’ plays, as they did not depict any activities related to girls. However, F6 and M7 were not entirely excluded from the study, as their drawings included representations of boys’ plays. Following this, the data from the drawings were systematically organized according to the established codes and categories. This organization ensured that the analysis was structured and aligned with the research objectives.

To ensure the reliability of the data analysis, coder reliability was employed. Five interview texts and drawings were randomly selected and coded independently by two separate coders. The agreement rate for the interview texts was calculated to be 95%, while the reliability level for the drawings was 92%. These high agreement rates indicate robust coder reliability (Miles et al., 2014). Furthermore, Stake (2005) identifies “particularity” and “thick description” as two defining features of case study research. Particularity refers to focusing on a specific and bounded phenomenon, while thick description involves providing a detailed and vivid account of the phenomenon under study. In this research, preschool children’s play perceptions were treated as the particular focus, and findings were presented with thick description, supported by direct quotations and drawings, to enrich the analysis. Additionally, to further enhance the validity and reliability of the study, representative quotations and selected drawings were incorporated into the analysis. This inclusion provides a more comprehensive and credible presentation of the findings.

### Ethical consideration

In this study, ethical guidelines outlined in “Ethical Research Involving Children [ERIC (Ethical Research Involving Children), 2023]” were strictly adhered to. Initially, the school administration and teachers were briefed about the research. An informed consent form was provided to the families of children who expressed a willingness to participate, and written consent was obtained from them.

### Findings

This section presents the findings derived from the analysis of children’s drawings and interviews. The results are organized into three main categories: *Children’s plays, children’s toys, and the motivations for children’s plays.*

#### Children’s drawings and opinions on girls’ and boys’ plays

**Figure 1:**
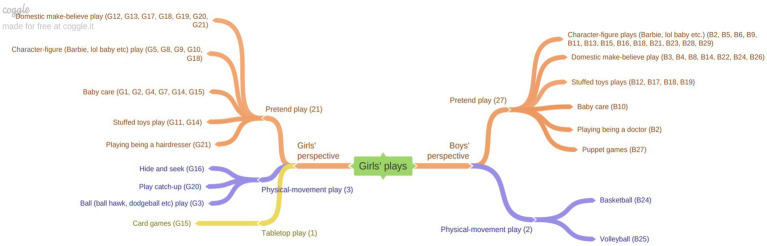
“Girls’ plays” from the perspective of girls and boys.

Figure 1 illustrates the types of play activities depicted in the children’s drawings that represent girls’ play.

**Figure 2:**
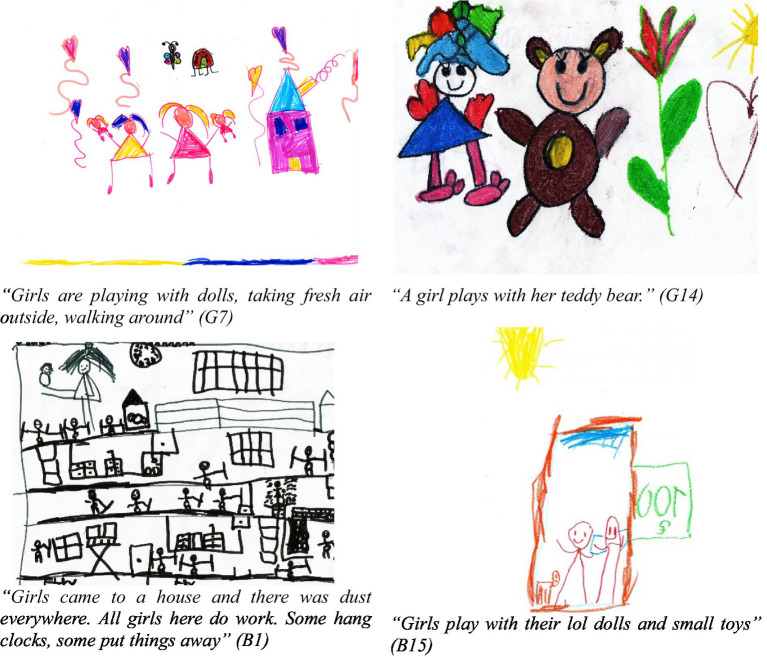
Children’s drawings of girl play.

The opinions of girls regarding play activities are categorized into three groups as shown in the figure above: Pretend play, physical movement play, and tabletop play. In the pretend play category, which girls identified as their most common type of play, the breakdown includes: Domestic make-believe play, baby care, character-figure play, stuffed toys play and playing as a hairdresser. Similarly, boys’ perceptions of girls’ play were also categorized under pretend play and physical movement play. The specific breakdown of the pretend play activities as described by boys differs slightly from the girls’ descriptions: Character-figure games, domestic make-believe play, playing with stuffed toys, playing as a doctor, baby care and puppet games. These findings indicate that, despite some differences in the specific activities reported, both girls and boys predominantly associate girls’ play with the category of pretend play, particularly domestic make-believe play, baby care, and character-figure games. Both genders expressed relatively few opinions about girls’ play in the category of physical movement games. Examples of children’s drawings and their accompanying explanations are provided below to illustrate these findings (Figure 2).

The following figure illustrates the results of the depiction of boys’ plays in children’s drawings.

**Figure 3:**
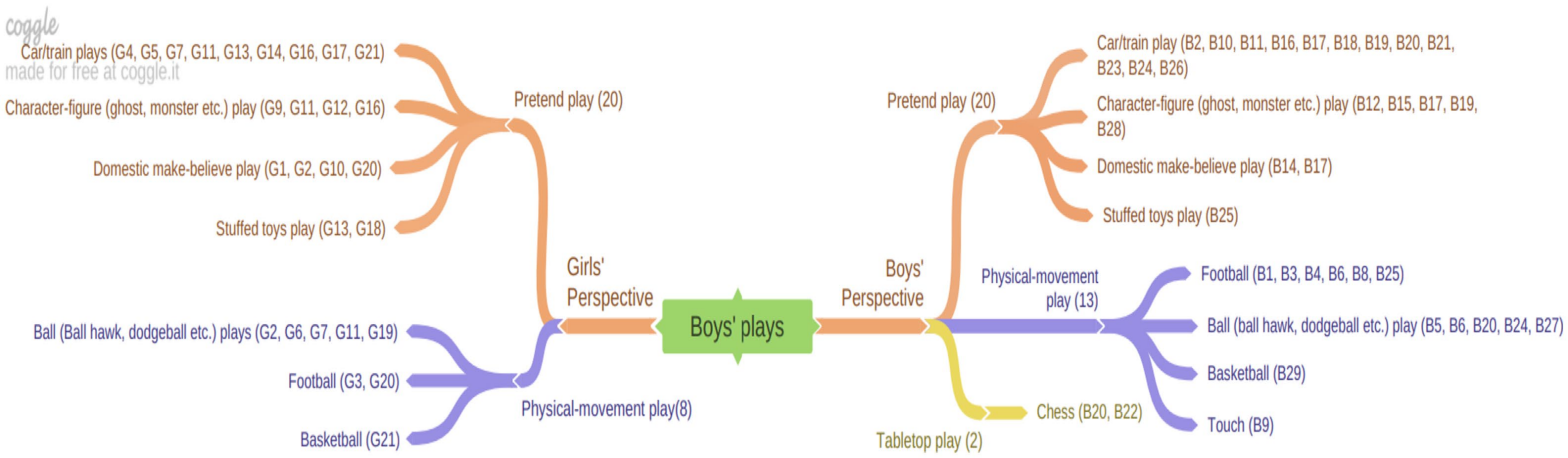
“Boys’ plays” from the perspective of girls and boys.

**Figure 4:**
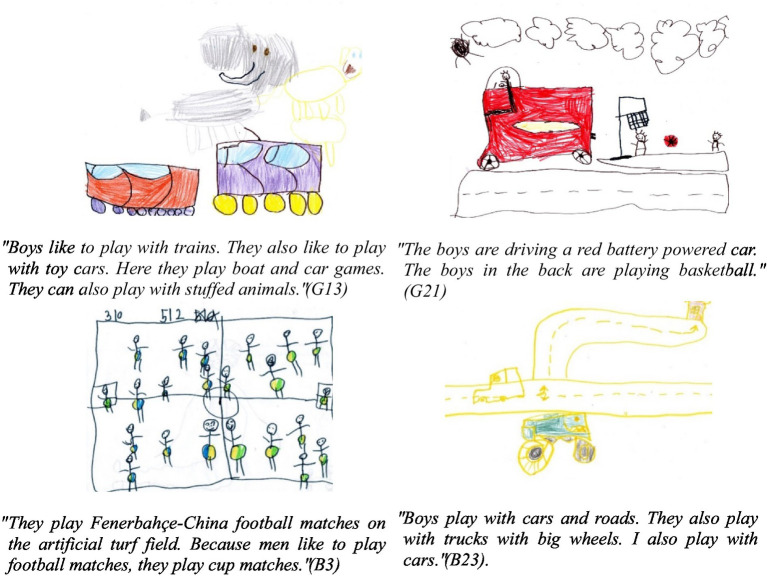
Children’s drawings of boy play.

Figure 3 categorizes girls’ perspectives on boys’ play into two primary categories: pretend play and physically active play. Within the pretend play category, which elicited the most responses from girls, the subcategories include car/train play, domestic make-believe play, character-figure play, and play with stuffed toy. Boys’ perspectives on boys’ play were similarly classified into pretend play, physical-movement play, and tabletop play. Within the pretend play category, boys expressed opinions on car/train play, character-figure play, domestic make-believe play, and play with stuffed toys, paralleling the responses of girls. Additionally, two boys mentioned that boys engage in chess, as part of their opinions on board games, a topic that was not addressed by girls. This data suggests that both girls and boys perceive that boys predominantly favor pretend play, particularly involving car/train play and character-figure play. Consistent with this finding, it was observed that car/train play was included in children’s views on boys’ play, unlike their perceptions of girls’ play (see Figure 1). Examples of children’s drawings and their corresponding opinions are provided below (Figure 4).

#### Children’s drawings and opinions on girls’ and boys’ toys

This section delineates the findings concerning the depiction of boys’ and girls’ toys as represented in children’s drawings, with an initial focus on the results of girls’ toys.

**Figure 5:**
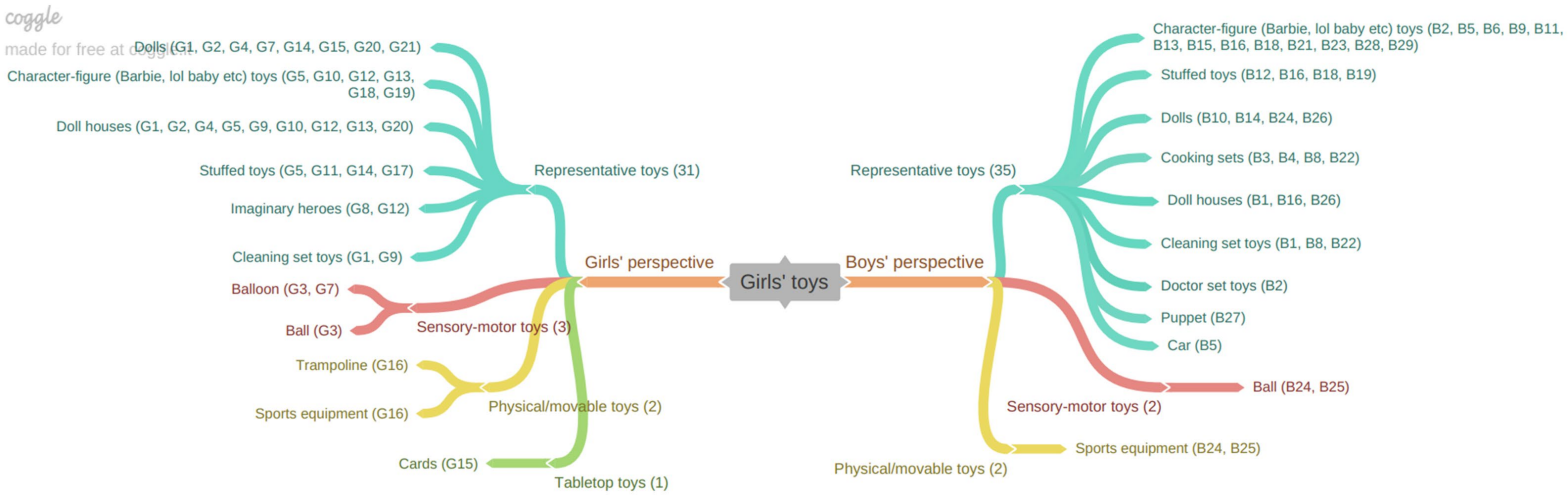
“Girls toys” from the perspective of girls and boys.

**Figure 6:**
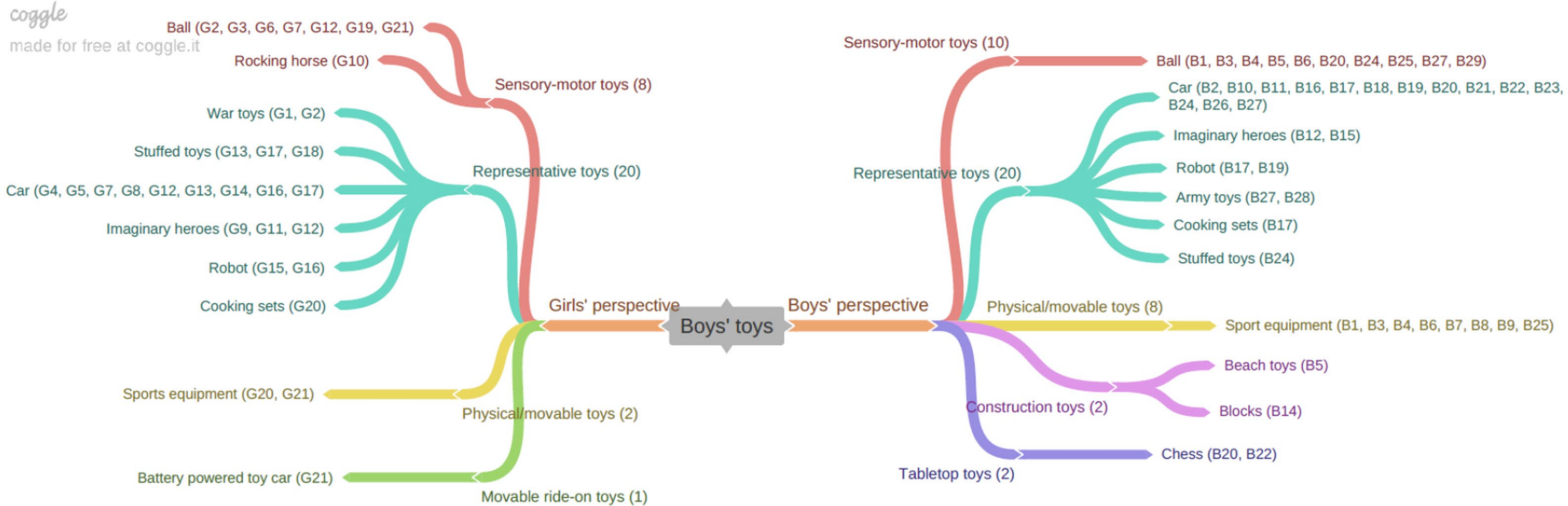
“Boys toys” from the perspective of girls and boys.

Figure 5 illustrates the categorization of girls’ perceptions of the toys they engage with, which are divided into four primary categories: representative, sensory-motor, physical/movable, and table-top toys. Representative toys, which garnered the most feedback from girls, include dolls, character-figure toys, dollhouses, stuffed toys, imaginary heroes, and cleaning tools. Similarly, boys’ perspectives on girls’ toys were also categorized into representative, sensory-motor, and physical/movement toys. Notably, unlike the girls, boys did not comment on table-top toys in their assessments. For the representative toys, boys perceived that girls predominantly play with character-figure toys, stuffed toys, dolls, cooking sets, dollhouses, cleaning set toys, doctor set toys, puppets, and cars. Overall, both girls’ and boys’ opinions are primarily centered around the “representative toy” category, with considerable alignment between the two groups. The subsequent figure presents children’s views on boys’ toys (Figure 6).

Girls’ perceptions of the toys that boys play with were classified into four categories: representative toys, sensory-motor toys, physical/mobile toys, and movable ride-on toys. Within the representative toys category, the most frequently mentioned items by girls included cars, imaginary heroes, stuffed toys, robots, army toys, and cooking sets. Similarly, boys also identified representative toys as predominantly favored by boys, with cars, imaginary heroes, robots, army toys, cooking sets, and stuffed toys being the most commonly cited items. Both girls and boys agreed that balls are the most frequently played with sensory-motor toys. However, in contrast to girls, boys also reported engaging with building construction toys and tabletop toys.

#### Children’s opinions on the motivations for boys’ and girls’ plays

The following figure presents the findings obtained from children’s views on the question, “Why do you believe girls engage in these types of play?” regarding girls’ plays.

**Figure 7:**
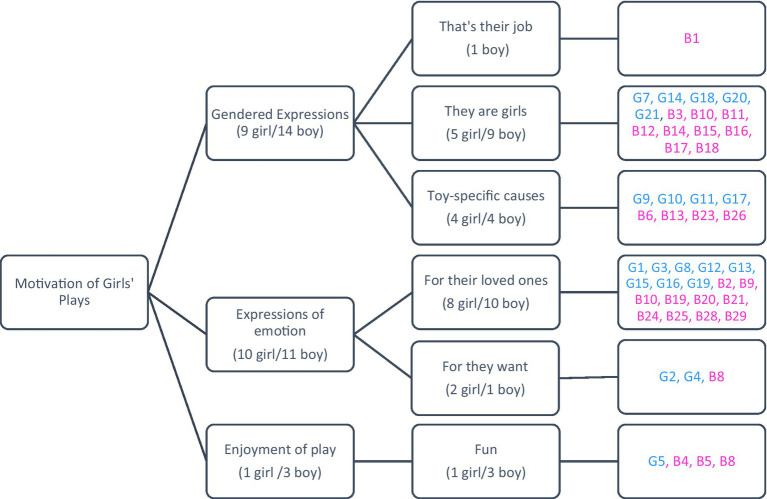
Motivation for girls’ plays from the perspective of girls and boys.

Figure 7 illustrates that children’s opinions were categorized into three main groups: gendered expressions, expressions of emotion, and enjoyment of play. Both girls and boys predominantly cited gendered expressions and expressions of emotion when explaining the motivations behind girls’ play activities. Notably, boys provided more comments related to gendered expressions compared to girls. The detailed opinions of both girls and boys on this topic are presented below.

Examples of girls’ responses categorized under gendered expressions include: *“Because they are girls, that’s what girls do”* (G7), *“Because … girls like LOL dolls. LOL dolls are for girls”* (G9), and *“Because domestic make-believe play is for girls. Girls love to play with home goods”* (G20). Examples of responses categorized under expressions of emotion are: *“Because they like to play with dolls”* (G1), *“Because they want to play with them”* (G2), and *“Because girls love to cook with toys”* (G13). An example of a response categorized under enjoyment of play is: *“Because they do it for fun”* (G5).

Examples of boys’ responses categorized under gendered expressions include: *“This is girls’ job (cleaning), they will earn money”* (B1), and *“They like to play with Barbie dolls because they are girls”* (B11). Examples of responses categorized under expressions of emotion are: *“They like to play with Barbie dolls and the doctor set”* (B2), *“Because they like to draw and play games”* (B9), and *“Girls like to play with Barbie dolls”* (B24). Examples of responses categorized under enjoyment of play include: *“They do it for fun”* (B4), and *“Because they are bored, they play for fun”* (B5).

The figure presented below illustrates the results derived from children’s responses to the question, ‘Why do you believe boys engage in these types of play?’

**Figure 8:**
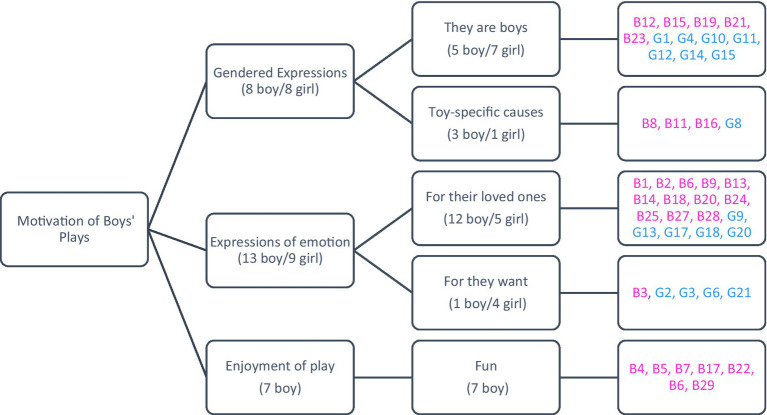
Motivation for boys’ plays from the perspective of girls and boys.

In Figure 8, children’s opinions are categorized into three groups: gendered expressions, expression of emotion, and enjoyment of play. Concerning the motivations behind boys’ play, both girls and boys identified gendered expressions and expressions of emotion as contributing factors. The specific opinions expressed by the children on this matter are detailed as follows:

Examples of the category “gendered expressions” provided by girls include: *“Boys like to play with cars because they have wheels and features. It’s just boys.”* (G8), and *“Because they are boys.”* (G12). In the category “expression of emotion,” girls offered examples such as: *“Because they like to play soccer.”* (G20). For boys, examples of “gendered expressions” include: *“Boys play with cars because cars are a boys’ game.”* (B11), and *“Because it is a boys’ game.”* (B15). In the “expression of emotion” category, boys provided examples like: *“Because boys like to play matches, cup matches.”* (B1), and “*Because I like it; boys like it a lot—guns, balls, cars.”* (B27). Regarding the “enjoyment of play” category, boys cited: *“Because they have fun.”* (B4), and *“Boys like soccer because it is very enjoyable.”* (B29). On the other hand, no girl expressed an opinion in the “enjoyment of play” category.

## Discussion

This study was conducted to determine the play and toy choices of Turkish children aged 48–72 months, with a focus on gender differences. In this context, the study investigated the play preferences, and toy usage inclinations of girls and boys toward both same-sex and opposite-sex activities, as well as their underlying motivations for engaging in these games. The study revealed that children’s views on girls’ play were predominantly categorized under “pretend play,” which includes activities such as domestic make-believe, baby care, and character-based games. However, both girls and boys indicated that girls engaged less in physically active games in their depictions. Conversely, opinions on boys’ play were largely classified under the “pretend play” category as well, but specifically involving car/train and character-based activities. In line with these findings, children associated car/train play more with boys than with girls and noted a greater inclusion of physically active play in boys’ games.

These observations reflect broader gender differences in early childhood play behaviors. Boys are generally inclined toward more active, rough, and competitive play, while girls tend to prefer quieter and more passive activities. Symbolic play among girls often centers around home-based themes, whereas boys’ symbolic play involves more physically energetic activities and superhero themes (Holland, 2003; Smith, 2009; Trawick-Smith, 2014). Gender differences are also evident in physical activities, with boys consistently engaging in games involving physical exertion from early childhood through adolescence (Campbell and Eaton, 1999; Frost et al., 2008; Lindsey and Colwell, 2003). Research indicates that boys and girls typically form same-sex peer groups during outdoor play and favor different types of physical activities. Boys often prefer playing in larger groups and engage in more competitive play, requiring more space than girls (Benenson et al., 1998; Thorne, 1993; Martin and Fabes, 2001). Additionally, a study by Lim (1998) in a Singaporean preschool found that girls engaged more in dramatic play with home-centered themes, while boys participated more in functional play involving motion-related activities such as running, jumping, and climbing. Similarly, Spanish preschool-aged children showed that girls engaged more in pretend play and boys in functional play (Yawkey and Alandrez-Dominquez as cited in Frost et al., 2008). Thus, the study underscores that both girls and boys exhibited a preference for play activities aligned with their gender roles, reflecting parallel opinions about each other’s play behaviors.

Another significant finding of our research is that children predominantly categorized their opinions about toys into the “representative toys” category for both boys’ and girls’ toys. Notably, there were observed differences in the types of representative toys preferred by each gender. Boys’ toys were primarily associated with cars, imaginary heroes, and military-themed items, while girls’ toys included dolls, character-figure toys, and dollhouses. Similarly, Blakemore and Centers (2005) found in their research that girls’ toys were associated with physical attractiveness, nurturance, and domestic skills, whereas boys’ toys were rated as violent, competitive, exciting, and somewhat dangerous. Additionally, children provide more feedback regarding sensory-motor toys than boys’ toys compared to girls’ toys in our research. These findings align with the observation that both girls’ and boys’ toy preferences are influenced by gender-based stereotypes, reflecting similarities in their views. It is evident that gender role behaviors impact the types of toys children prefer. Consistent with Pellegrini and Perlmutter’s research (as cited in Smith, 2009), gender roles also affect how materials are utilized in boys’ and girls’ play.

Literature suggests that children’s play behaviors exhibit gender-based tendencies even before they fully develop an understanding of gender roles or stereotypes. For instance, children often show a preference for gender-stereotypical toys well before they become conscious of gender differences. Girls tend to favor dolls, whereas boys are more inclined toward trucks or building blocks (Bee and Boyd, 2009; Zahn-Waxler et al., 2008). These gender-based variations in play and toy preferences can be observed as early as 18 months and become more pronounced around age three (Golombok et al., 2008). Moreover, in their three-stage study, Martin et al. (1995) demonstrated that children approximately 58 months of age not only identify toys that are suitable for their gender but also accurately infer the toy preferences of children of the opposite gender. Building on this work by Martin and colleagues, Lam (2023) found in her recent research that infants, on average 40 months old, tend to predict peers’ liking for -novel- nonstereotyped toys based on their own and peers’ gender as a form of stereotype construction.

During the preschool years, boys typically prefer toys such as repair tools, vehicles, swords, and guns, while girls gravitate toward dolls, tea sets, and household items (Dunn and Hughes, 2001; Martin et al., 1990; Zosuls et al., 2009). Boys often engage in active play involving building blocks, cars, and toy vehicles, whereas girls are more inclined toward seated activities like drawing, modeling with clay, and playing with dolls (Frost et al., 2008). A study by Gavrilova et al. (2023), investigated the influence of sociodemographic factors such as gender, children’s age, mother’s education level, and number of siblings on toy preferences among three to four-year-olds, it was found that gender and the number of siblings were significant predictors of toy preferences. The study also noted that boys tended to select more detailed toys compared to girls. These findings underscore the importance of gender as a variable in children’s toy choices and its impact on their preferences. Similarly, Kislev and Saguy (2025), using the Gendered Toy Choice (GTC) measure, found that parents were more likely to avoid counter-stereotypical toys for their sons than for their daughters. Their results demonstrated that, regardless of cultural context, parents more strongly avoided such toys for boys, reflecting the influence of prescriptive and proscriptive gender norms. These findings are consistent with the results of the present study and further support the conclusion that children’s play and toy preferences are shaped not only by individual inclinations but also by parental practices and broader cultural influences. The categorization of toys as specifically “boys’ or girls’ toys” by children in this study suggests that these distinctions may reflect socially learned responses shaped by their environment and interactions, rather than gender-based labels and stereotypes as fixed constructs. In another study, Todd et al. (2018) conducted a meta-analysis of observational studies to determine the variables that predict toy preferences of children aged 1–8 and found that both girls and boys play with toys appropriate to their gender. In addition, in the same study, they found that the presence of an adult, study context, geographical location of the study, publication date, child’s age, or the inclusion of gender-neutral toys did not affect children’s toy preferences. As a result, they suggested that this consistent pattern of selecting gender-appropriate toys, despite variations in methodological approaches, testing contexts, and the ages of the children, might have a biological basis (Todd et al., 2018). In a separate study, Todd et al. (2017) showed the potential biological underpinnings of gender-related toy preferences. Conducted in England, this study included 40 infants aged 9 to 17 months, who were beginning to exhibit toy preferences; 29 infants aged 18 to 23 months, who had made significant strides in gender knowledge; and 32 infants aged 24 to 32 months, who demonstrated advanced gender knowledge. The findings revealed that all three age groups exhibited stereotypical toy preferences, suggesting a possible biological predisposition influencing these preferences.

Another key outcome of our study is that both girls and boys identified three primary motivations for play: gendered expressions, expressions of emotions, and the enjoyment derived from play. Children often explain their motivations for engaging in certain activities by emphasizing the emotional aspects and the pleasure associated with the play experience. This indicates that while children categorized play based on gender, they also valued and recognized the intrinsic enjoyment provided by the play process. Play serves as a crucial context in which children experience and interpret a range of emotions, including fun, satisfaction, surprise, curiosity, and expectation. It is a primary medium through which children learn to express, process, regulate, and utilize emotions adaptively (Frost et al., 2008; Russ, 2004). Consequently, children’s views that they engage in play because they enjoy it, desire it and find it fun are consistent with existing literature. However, the study also revealed that children attributed motivations for both girls’ and boys’ play to gendered expressions, such as the belief that certain activities or toys are inherently gendered. This finding suggests that children have developed an understanding of gender identities, both their own and those of the opposite sex, and reflect this understanding in their motivations for play. It implies that cultural and environmental factors significantly influence these perceptions. Children learn about gender differences in play from family members, peers, and mass media, with their gender-congruent play being shaped by the cultural context in which they live (Bornstein et al., 1999). Across culture, parents aim to raise their children as competent members of their cultural groups, and therefore guide their behaviors to align with cultural norms. Play is often regarded by parents as a tool through which they can teach children culturally appropriate behavioral patterns (Le et al., 2008; Lin et al., 2019). In a study examining Turkish mothers and children’s symbolic play behaviors, it was found that mothers most frequently participated in play as managers. This tendency was explained by their desire to convey cultural messages to their children through the themes they introduced, the degree of autonomy they allowed, and the roles they assumed during play (Aksoy et al., 2022). For instance, parents may reinforce gender-specific play by praising activities that align with gender norms or discourage non-gender-specific activities by removing children from such games (Pasterski et al., 2005). Klemenović (2014) points out that parents tend to be more involved in children’s play by giving more positive verbal responses when children play with toys that are stereotypes of their gender. Thus, the tendency for children to justify their play preferences based on gender roles reflects the impact of cultural socialization and parental expectations on their play behaviors.

## Conclusion

In line with the results, girls’ and boys’ views on play and toys show gender-related differences. Nonetheless, children’s opinions are parallel. An essential result of the study is that both girls and boys expressed similar views about their own play and each other’s play. Children’s gendered expressions about the motivations for their play preferences suggest that their views may reflect socially learned responses shaped by their environment and interactions. Determining children’s gender-based play behaviors in early childhood is crucial to obtaining information about children’s development and implementing intervention programs when necessary.

The findings of this study carry important implications for early childhood education and parenting practices. Educators can support more equitable play opportunities by offering a wider range of toys and activities that are not limited by gender stereotypes. Encouraging children to explore non-stereotypical play options may contribute to their cognitive, social, and emotional development. Parents and caregivers can also play a critical role by modeling inclusive attitudes, praising children’s diverse play choices, and providing access to toys and activities that foster creativity and cooperation rather than reinforcing rigid gender roles. These practices can help create a more balanced and less stereotyped play environment, promoting healthier developmental outcomes for both boys and girls.

## Limitations and future directions

While this study offers theoretical and practical contributions to the literature, several limitations should be addressed in future research. One notable limitation is the relatively small sample size of participants. The study was conducted with a limited number of children, which may affect the generalizability of the findings. Given the prevailing stereotypes regarding children’s play and toy preferences, there is a need for further investigation into the nature of gender differences in children’s play behaviors from infancy onward. To gain a more comprehensive understanding, future research should employ a variety of methods and involve larger sample sizes. Additionally, exploring gender-related play and toy preferences across different cultures, ethnicities, and socioeconomic backgrounds could provide valuable insights.

## Data Availability

The raw data supporting the conclusions of this article will be made available by the authors, without undue reservation.
